# What are the barriers and facilitators of clozapine use in early psychosis? A survey of UK early intervention clinicians

**DOI:** 10.1038/s41537-023-00353-0

**Published:** 2023-04-28

**Authors:** Ebenezer Oloyede, Graham Blackman, Bethany Mantell, Eleanor Harris, Julie Williams, David Taylor, James MacCabe, Philip McGuire

**Affiliations:** 1 South London and Maudsley NHS Foundation Trust, London, UK; 2 Department of Psychosis Studies, Institute of Psychiatry, Psychology & Neuroscience, King’s College London, London, UK; 3 Department of Psychiatry, University of Oxford, Warneford, UK; 4 Health Service and Population Research Department, Centre for Implementation Science, King’s College London, London, UK; 5 NIHR Biomedical Research Centre for Mental Health South London and Maudsley NHS, London, UK

**Keywords:** Schizophrenia, Psychosis

## Abstract

Clozapine is the most effective medication for treatment-resistant psychosis, but evidence points to substantial underuse, especially within early intervention psychosis (EIP) services. We explored clinicians’ views on perceived barriers and facilitators to offering patients clozapine within EIP services. A cross-sectional survey was distributed electronically to clinicians practising in EIP services across England. A mixed methods approach was used to assess barriers to clozapine, and attitudes and opinions concerning clozapine underutilisation. Based on the barriers identified in the literature, clinicians were asked to rate each one (scale:1-7) based on importance, with a higher score indicating higher importance. Clinicians were also asked open-ended questions on barriers to clozapine and how access can be improved in EIP services. Quantitative data were analysed using descriptive and inferential statistics, and qualitative responses were analysed thematically. One hundred and nineteen EIP clinicians from 35 services in England completed the survey. In total, 37% (*n* = 45) of clinicians perceived that clozapine was under-prescribed in their EIP service. The most important barrier to utilising clozapine were patient concerns with side effects, followed by monitoring requirements and clinician concerns with side effects. Thematic analysis identified 17 perceived barriers, which were grouped into three major themes: administrative (5 subthemes), clinician-related (6 subthemes), and patient-related (6 subthemes). Perceived facilitators to improving clozapine use were greater training, improved resources, and optimised monitoring. The main barriers to clozapine in EIP services, as identified by clinicians, are patient concerns regarding side effects and monitoring requirements. Identified facilitators for improved clozapine use include clinician training, improved resources, guidelines, and point-of-care testing.

## Introduction

Approximately one in three patients with a diagnosis of schizophrenia are considered “treatment-resistant”, defined as having persistent psychotic symptoms despite sequential trials of at least two antipsychotics^1^. Clozapine is the only evidence-based antipsychotic for treatment-resistant psychotic disorders^2^. However, despite substantiative empirical evidence for its clinical efficacy, clozapine remains underutilised^2–4^. At a patient level, the underutilisation of clozapine in treatment-resistant psychosis (TRP) is associated with decreased interpersonal and occupational functioning, decreased quality of life and increased caregiver burden and hospitalisation^5,6^. While clozapine underuse is likely to be multifactorial, one purported factor is clinician perception^7,8^. Specifically, studies have suggested that clinicians may overemphasis the negative aspects of clozapine treatment, particularly in comparison to patient views^9^. Systematic reviews exploring broader barriers for clozapine underuse have identified three broad domains, namely patient-related (e.g. mandatory haematological monitoring), clinician-related (e.g. prescribing experience) and administrative (e.g. service fragmentation) barriers^10,11^. Furthermore, previous surveys of psychiatrists working in adolescent and old age services within the United Kingdom (UK) have highlighted practical difficulties in prescribing and monitoring clozapine^12,13^. In contrast, the barriers to clozapine use in early intervention in psychosis (EIP) services to date has been relatively unexplored.

In the UK, EIP services provide a range of psychological, social, and pharmacological interventions to promote recovery from a psychotic episode^14^. Increasing evidence have shown that TRP is prevalent in almost a quarter of people with first episode psychosis (FEP) in EIP services^5,15^. Recently, greater emphasis has been placed on improving access to clozapine for patients with TRP across EIP services in England^16,17^. This is based on evidence that a delay in offering clozapine reduces the likeliness of treatment response^18^. Despite the aforementioned EIP service priority within the UK, prescription rates of clozapine in EIP services remains very low^3^. Health care providers have a critical role in improving clozapine utilisation rates in EIP services. We therefore sought to identify their views on perceived barriers and potential facilitators to clozapine use.

## Methods

This was a mixed-methods study using a cross-sectional survey of clinicians in EIP services across England to determine the attitudes towards clozapine use in EIP services and the perceived barriers and facilitators to clozapine treatment.

### Survey Instrument

An online survey was developed by a multidisciplinary team consisting of psychiatrists, pharmacists, psychologists and occupational therapists following guidelines for designing electronic survey designs^19^ and has previously been described^20^. In brief, the survey comprised of 15 items divided into three areas: respondent demographics (7 items) (*e.g. what organisation do you work for?*); attitudes toward clozapine use in EIP services (5 items) (e.g., *do you feel patients prefer taking clozapine compared to other antipsychotics?*); and perceived barriers and facilitators to clozapine use in EIP services (3 items) (e.g*. what would be helpful in facilitating clozapine initiation in your EIP service?*). Clinicians were asked to score six purported barriers to clozapine use within EIP services on a scale of 1 to 7 (1 representing the *least* important and 7 representing the *most* important). The potential barriers were selected based on a review of the literature^9,10,21–23^. Clinicians were then asked to categorise clozapine prescribing rates in patients eligible within their EIP service as either: (i) ‘overprescribed’ (ii) ‘adequately prescribed’, (iii) ‘under prescribed’ or (iv) ‘unclear’. Clinicians also answered two qualitative questions (i) what they thought were the current barriers to clozapine use and (ii) how clozapine offer can be improved in EIP services. The survey was completed online using Qualtrics XM (Provo, Utah, USA). A pilot study of 60 clinicians was initially conducted within a large mental health provider (South London and Maudsley NHS Foundation Trust) to assess item range, variance, content and clarity. The final version of the survey took a median of 7 minutes (interquartile range 5 to 11 minutes) to complete (survey available on request).

### Recruitment and ethical considerations

Clinicians were recruited through EIP services across England. One hundred and nine EIP services in England were contacted via email and 35 services disseminated the survey to their clinicians. The survey was accessible for 12 weeks. Ethical approval was obtained from King’s College London (*MRSU-20/21-25297)* and individuals gave informed consent prior to beginning the survey.

### Analysis

Quantitative analysis was conducted using *R*^24^. Survey items concerning the importance of perceived barriers for clozapine use in EIP services were described using means and standard deviations. A Mann-Whitney *U* test was used to compare identified barriers between prescriber’s and non-prescribers. The threshold for statistical significance was *p* ≤ 0.05. Bonferroni correction was performed to adjust for multiple comparison. Categorical responses were summarised using percentages. Open-ended responses regarding barriers and facilitators to clozapine use were analysed thematically^25^. Thematic categories were constructed inductively from response data by two independent researchers (EO and BM). They then met to compare themes and subthemes and derive consensus. Following creation of these themes and subthemes, the two researchers coded each response independently then met to discuss any discrepancies and derive consensus.

## Results

### Sample characteristics

E-mail invitations were sent to 35 EIP services, comprising approximately 700 clinicians. Of these, 193 accessed the survey and 123 (approximately 18% of all eligible clinicians) completed the survey from the 35 EIP services and were included in the analysis. Respondents consisted of 63 females (67%) and the mean age was 44 years. The most common professions were nurses (50%), doctors (19%) and psychologists (9%). In total, 31 (32%) of responders were prescribers. Participant characteristics are displayed in Table 1.

**Table 1:** Characteristics of Survey Respondents.

Respondent characteristics	Total Sample (*n* = 123)	Prescribers (*n* = 37)	Non-prescribers (*n* = 86)
Gender *n* (%)			
Female	82 (66.7)	20 (54.1)	62 (72.1)
Male	40 (32.5)	16 (43.2)	24 (27.9)
Prefer not to say	1 (0.8)	1 (2.7)	0 (0.0)
Age (mean years, SD)^a^	43.8 (11.5)	48.3 (8.6)	42.1 (12.1)
Self-reported profession^a^ *n* (%)			
Nurse	63 (51.2)	6 (16.2)	57 (66.3)
Doctor	24 (19.5)	24 (64.9)	0 (0.0)
Psychologist	8 (6.5)	0 (0.0)	8 (9.3)
Social Worker	8 (6.5)	0 (0.0)	8 (9.3)
Other	12 (9.8)	0 (0.0)	11 (12.8)
Consultant Psychiatrist	6 (4.9)	6 (16.2)	0 (0.0)
Pharmacist	2 (1.6)	0 (0.0)	2 (2.3)
NHS Regional Team *n* (%)			
East of England	10 (8.1)	2 (5.4)	9 (9.3)
London	44 (35.8)	14 (37.8)	30 (34.9)
Midlands	14 (11.4)	3 (8.1)	11 (12.8)
Northeast and Yorkshire	9 (7.3)	5 (13.5)	4 (4.7)
Northwest	8 (6.5)	5 (13.5)	3 (3.5)
Southeast	23 (18.7)	3 (8.1)	20 (23.3)
Southwest	15 (12.2)	5 (13.5)	10 (11.6)
Clinical experience *n* (%)			
0–5 years	33 (26.8)	2 (5.4)	31 (36.0)
6–10 years	22 (17.9)	1 (2.7)	21 (24.4)
11–20 years	31 (25.2)	13 (35.1)	18 (20.9)
21+ years	37 (30.1)	21 (56.8)	16 (18.6)

^a^Missing data in 1 respondent.

### Clinician attitudes and clozapine barriers

Based on their experience of working in an EIP service, 38% of clinicians responded that clozapine was ‘adequately prescribed’ and 37% of clinicians responded that clozapine was ‘under prescribed’ and 2.3% felt that it is ‘overprescribed’. A further 23% of respondents were ‘unclear’.

**Fig. 1:**
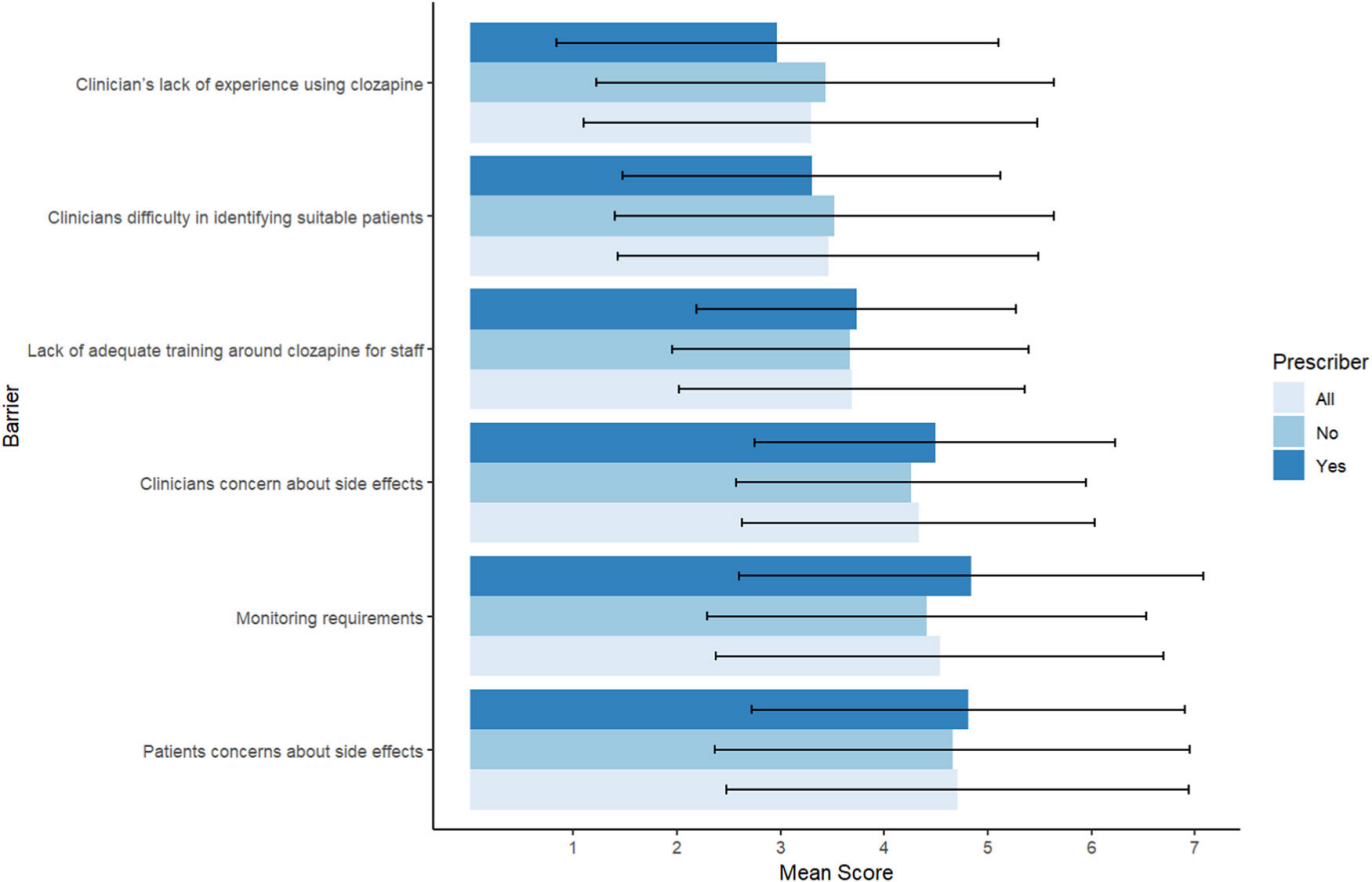
Perceived barriers to clozapine treatment. Mean item scores and standard deviations reported. A higher score indicates a more important perceived barrier according to clinicians in EIP services (score ranges: 1–7).

The most important perceived barrier according to clinicians was ‘patient concerns with side effects’ (mean = 5.7, SD = 1.6), followed by ‘monitoring requirements’ (mean = 5.4, SD = 1.7) and ‘clinician concerns with side effects’ (mean = 4.4, SD = 1.7) (see Fig. 1). The least important barrier was ‘clinicians’ difficulty in identifying suitable patients’ (mean = 5.7, SD = 1.5). There was no significant difference between prescribers and non-prescribers for identified barriers.

### Qualitative results

#### Barriers

Three major themes were identified regarding perceived barriers to clozapine use (illustrative quotes in Table 2). The most frequently mentioned theme was administrative barriers (*n* = 35, 41%), with the subthemes of blood monitoring, clozapine initiation, patient information, resources and treatment pathways. The second most common theme was patient-related barriers (*n* = 33, 39%), with the subthemes of adherence, blood monitoring, family and carer attitudes, patient attitudes, side effects and stigma. Finally, for clinician-related barriers (*n* = 17, 20%), the subthemes were clinician attitudes, formulation, prescriber confidence, side-effects, treatment pathway and TRP identification.

**Table 2:** Representative quotes from thematic analysis on barriers to clozapine access in EIP services.

Barriers to clozapine access		
Themes	Subtheme	Clinician quotes
Administrative	Blood monitoring	“Monitoring when patients are in full time employment and being able to access services out of office hours” “Travel commitments to the clozapine clinic from distant rural / deprived areas”
	Clozapine initiation	“No community titration team. Patients have to come into hospital to be titrated currently.”
	Patient information	“The patient information sheets do not give any encouragement or evidence for how clozapine is effective or helpful, but they primarily focus on listing all the possible side effects and risks with some leaflets including disturbing images as examples of what they refer to ie weight gain showing someone morbidly obese, or someone unable to open their eyes for sedation or excessive drooling for hyper salivation. There is nothing in the patient or carer information“
	Resources	“In areas where there is no clozapine clinic, most often rural/semi-rural areas, some GP’s refuse to allow monitoring blood tests in their surgeries leading to patients having to travel to hospital each time they need a blood test. This can be anxiety provoking and costly in terms of money and time for the patient. Management level agreements with these practices would be helpful.”
	Treatment pathways	“Clear pathways for community patients over large geographic area.”
Patient-related	Adherence	“Compliance with oral medication and the risks around service users forgetting or discontinuing inappropriately“
	Blood monitoring	“Patient’s ability to attend regular blood tests and teams’ availability to facilitate this” “Patient not willing to have blood test or have it less frequently”
	Family and carer attitudes	“Carers concerns; and third party reports around the side-effects/monitoring requirements”
	Patient attitudes	“Stigma associated with taking clozapine”
	Side effects	“Concerns about giving clozapine to younger teenagers with side effects that may not reverse.”
	Stigma	“Clozapine is referred to as a treatment option for clients regarded treatment resistant. There should be consideration for clients, in being referred to as treatment resistant and what this means for the individual themselves, the negative stigma (including self-stigma) of being almost untreatable.” “Stigma associated with taking clozapine”
Clinician-related	Clinician attitudes	“Clinician’s saying clients are needle phobic so won’t prescribe. There is a solution for this!” “Clinician’s hesitancy in starting clozapine due to patients previously being non-compliant with oral treatment”
	Formulation	“Concerns that diagnosing a psychosis as treatment resistant can mask underlying psychological issues that in my experience have often not been identified or adequately addressed. This often includes historic trauma and traumatic loss and bereavement.”
	Prescriber confidence	“I think there is a lot of anxiety about prescribing for prescribers that lack experience in clozapine.”
	Side-effects	“Significant side effects and monitoring for service user.”
	Treatment pathway	“It has often been the case that clients admitted to acute wards are trialled on numerous antipsychotics, contrary to recommended guidelines. Inpatient psychiatrist will often make their own decisions regarding medication treatment and will remain resistant to any input from the community team regarding the consideration of other medication options, i.e., clozapine” “Reputation of clozapine as a last resort when clinicians have given up on all else”
	TRP identification	“Identifying treatment resistant as opposed to non-adherence”

Treatment resistant psychosis (TRP).

#### Facilitators

Eight major themes were identified regarding how to facilitate clozapine use in EIP services (illustrative quotes in Table 3). The most frequently mentioned theme was resources (*n* = 31, 26%), followed by patient information (*n* = 24, 20%), training (*n* = 16, 14%), clinical pathways (*n* = 15, 13%), monitoring (*n* = 11, 9%), guidance for clinicians (*n* = 9, 8%), side effects (*n* = 8, 7%) and perspectives on clozapine treatment (*n* = 4, 3%). For resources, subthemes were facilitated clozapine initiation, community resources, dedicated service, monitoring, staffing and TRP identification. For patient information, subthemes were clozapine benefits and risks, patient-friendly information, testimonials and counselling. For training, subthemes were benefits and risks, family and carer training, accessibility, improving clinician knowledge and confidence. For clinical pathways, subthemes were TRP identification, timely and proactive monitoring of non-clozapine treatment response, communication between services, timely and proactive clozapine offer and a dedicated service. For monitoring, subthemes were optimised monitoring, point of care (POC) blood testing and antipsychotic adherence. For the theme guidance for clinicians, subthemes were clozapine initiation and TRP identification. For the theme side effects, subthemes were physical health, proactive management and interventions. For the theme perspectives on clozapine treatment, subthemes were addressing stigma and clinician attitudes. Illustrative quotes can be found in Table 3.

**Table 3:** Representative quotes from thematic analysis on improving clozapine access in EIP services, Treatment-resistant psychosis (TRP).

Facilitators to clozapine access		
Theme	Subtheme	Clinician quotes
Resources	Facilitate clozapine initiation	“Better resourcing services would allow clinicians to more thoroughly discuss treatment options including different medications with service users who could then be supported to make informed decisions”
	Community resources	“An easy way of recording what treatments have been tried and why they were unsuccessful. More infrastructure for supporting patients, carers and staff to initiate clozapine in the community”
	Dedicated service	“It’s a very long-winded and bureaucratic procedure that takes a lot of time to set up.If there was a dedicated team within each trust to do all the setting up and admin it would help enormously.”
	Monitoring	“Support with the monitoring requirements - service is under staffed and struggling”
	Staffing	“Dedicated pharmacy support and advice for suitable patients”
	TRP identification	“More access to medic reviews”
Patient information	Clozapine benefits and risks	“Up-to-date evidence that the benefits outweigh the risks, especially for the younger age group.”
	Patient-friendly information	“Simplified literature /media to show the therapeutic benefits”
	Testimonials	“Testimonial videos” “More positive first person narratives from people on clozapine to offer to patients and their families”
	Counselling	“Patient counselling”
Training	Clozapine benefits and risks	“More education about the benefits!”
	Family and carer training	“Having training for family members”
	Accessibility	“Increased access to training to help people become more informed about the evidence base for clozapine”
	Improve clinician knowledge	“More knowledge for the clinician/practitioner”
	Improve clinician confidence	“Well informed prescribers to confidently have good discussion and communication with clients”
Clinical pathway	TRP identification	“To have a designated community pathway for initiation and monitoring.”
	Timely and proactive monitoring of non-clozapine treatment response	“Keeping a log of individual timelines for initiating antipsychotic treatments, better education of MDT; focussed review of timelines of antipsychotics tried and good documentation of the nature of the therapeutic response to antipsychotics, early education of patients and carers”
	Communication between services	“Better communication and collaboration between inpatient and community teams and with carers and patients”
	Timely and proactive clozapine offer	“Robustly trialling previous antipsychotics in proper sequence then offering clozapine within a reasonable time frame”
	Dedicated service	“To have a designated community pathway for initiation and monitoring.”
Monitoring	Optimised monitoring	“Blood tests done at home with immediate results as weekly tests at a clinic put people off and is expensive”
	Point of care blood testing	“Making the monitoring easier for patients - blood test put people of - I understand there is equipment that can give instant results every EIS should have this” “Point of care testing for less intrusive and instant feedback”
	Antipsychotic adherence	“During inpatient admissions, monitoring compliance”
Guidance for clinicians	Clozapine initiation	“More information/guidance, upskilling staff confidence/knowledge” “Knowing when to say that a drug is not working… should they have no symptoms?”
	TRP identification	“Clarity over what treatment-resistance means”
Side Effects	Physical health	“Offering a robust healthy living programme”
	Proactive management	“Side effect management”
	Interventions	“Better ways to manage side effects”
Perspectives on clozapine treatment	Addressing stigma	“Culture change around perception of clozapine” “Reduced stigma and better more accessible information about the pros of taking clozapine”
	Clinician attitudes	“A more positive attitude from prescribers”

## Discussion

We undertook a national survey of clinicians practising in EIP services in England to examine the perceived barriers and potential facilitators to clozapine use. Over a third of clinicians reported that clozapine was under-prescribed. Based on thematic analysis, we identified 17 perceived barriers that could be broadly grouped into administrative, clinician-related and patient-related themes. Clinicians considered patient concerns about side effects as the most substantial barrier to clozapine, followed by monitoring requirements. Facilitators for improved clozapine use in EIP services included more guidance for clinicians, followed by optimised monitoring and education and training interventions.

To the best of our knowledge, this is the first study to have explored the perceived barriers and facilitators of clozapine use exclusively in EIP services. Consistent evidence has demonstrated widespread underuse and variation of clozapine prescribing and this is seemingly more prevalent in EIP services^3^. Barriers to improved clozapine treatment have been previously studied and can be broadly divided into patient, clinician and administrative^10,11,26,27^, which our survey also identified. A previous review on the barriers to the use of clozapine highlighted insufficient prescriber training; concern over rare, but serious side effects (including agranulocytosis and myocarditis); frequent blood tests; relatively high administrative burden; and side effects (including weight gain, sedation, and hypersalivation) as prominent barriers^10^. Another review on prescriber and institutional barriers and facilitators of clozapine similarly identified side-effects, monitoring requirements and clinician prescribing experience as major determinants of under-prescription^11^. We found that the barriers to clozapine use specifically in EIP were similar to the aforementioned systematic review, where the authors argued that the lack of practical knowledge with prescribing clozapine was the most likely cause of the negative beliefs surrounding the medication^10^.

Thematic analysis revealed perceived facilitators to improved clozapine use in EIP services. These were increased clinician training, addressing clinician attitudes towards clozapine and improved resources (including use of POC testing). Requests for education and training interventions to promote safe and effective use of clozapine have been made for years^23,28,29^. Nevertheless, there is currently no consistent approach to the training of clinicians for clozapine management. The implementation of training and learning from experienced clozapine prescribers are likely to increase clinician confidence^11,20,22,30^. Consistent with the broader literature, addressing clinician attitudes towards clozapine was considered an important facilitator by EIP clinicians^11^. Indeed, previous studies have demonstrated that clinicians tend to overstate patient reluctance with clozapine use^31^. For example, in a clinician survey by Hodge and Jespersen (2008), 52% of respondents estimated that patients were unhappy with the mandatory blood tests, compared to only 19% of the patients when directly asked^9^. Moreover, clinicians often state concerns with adherence to clozapine, despite evidence suggesting that adherence is comparable to other antipsychotics^32^. Consistent with existing literature, our study identified improved resources, and in particular use of POC testing, as potential facilitators to improved clozapine use^10^. Encouragingly, recent work within the UK has demonstrated the effective use of POC testing, including capillary clozapine levels and neutrophil monitoring^33,34^. The implementation of education and training interventions across EIP services in England alongside wider use of POC testing are potentially cost-effective methods of addressing clinician-related barriers to clozapine. Addressing administrative and structural barriers to improved clozapine, such as ensuring access to local clozapine outpatient clinics for patients, should also be a priority as this would likely have a synergistic effect on other interventions, such as pharmacy support and POC testing. Of note, in the Netherlands, the discontinuition of regular blood tests and institutional pressure (such as regular auditing or mandating detailed action plans for centres with low prescription rates) yielded the second highest recorded increase in clozapine use^35,36^. Notably, since these changes by the Dutch Clozapine Collaboration Group, the average outpatient prescription rates of clozapine in the Netherlands have increased by 88% between 2004 and 2019. Moreover, organisational changes such as education and quality improvement initiatives have led to significant increases in clozapine use in clinical services in New Zealand and New York^37,38^. Similar interventions in EIPs in the UK could conceivably lead to similar improvements.

### Clinical implications

This study highlights several key areas of potential intervention to potentially improve clozapine treatment access for individuals with TRP in EIP services. Our study suggests that ensuring service provision is sufficient (including an accessible clozapine clinic or equivalent service) may remove a key administrative barrier to initiating and maintain clozapine treatment. Access to expertise, such as a “hub and spoke” model, may also help clinicians with clozapine management^3^. This is supported by evidence that most clinicians view clozapine prescribing as a burden^39^. Reducing the perceived cumbersome nature of clozapine monitoring by minimising the degree of physical health monitoring (whilst maintaining safety standards) through evidence-based guidelines alongside use of POC testing could reduce barriers to clozapine use, improve patient satisfaction and overall increase clozapine utilisation^40^. Our data highlights the potential benefit of ensuring professionals working within EIP services have the opportunity to develop their skills, knowledge and confidence in managing and prescribing clozapine treatment through education and training interventions, such as through e-learning^20^. This may also increase clinician confidence when communicating with patients and their families and caregivers around clozapine treatment, however studies are required to confirm this. Addressing misconceptions around clozapine use through training and patient testimonials is equally important. For example, delegating clozapine monitoring to advanced nurse practitioners did not improve clozapine prescription rates in a randomised trial in the Netherlands^41^. While the study was likely underpowered to detect a difference, the authors suggested that the lack of improvement may have been due to misconceptions about clozapine’s therapeutic potential among prescribing psychiatrists. In addition to education and training for clinicians, respondents suggested potential benefit of similar interventions for patients, family and carers. Future studies should investigate service user views on facilitators to improved clozapine use in EIP services.

### Strength and limitations

To our knowledge, this is the first study to explore the perspectives of EIP clinicians on barriers and facilitators for increased clozapine use in EIP services. Further strengths of this study include the wide geographical coverage, and inclusion of a range of healthcare professionals. Another strength is the inclusion of both non-prescribers and prescribers. While prescribers decide treatment plans in collaboration with the patient, non-prescribers, such as mental health nurses, are often assigned the key role of coordinating patient care. Care coordinators maintain contact with patients, produce care plans and typically attend medical reviews with clients and prescribers. As such they are well-placed to flag which patients may be good candidate for a trial of clozapine.

A major limitation of our study is that we were unable to ascertain a precise number of the total number of clinicians from which we sampled. Our study is also limited by the low response rate of around 17%, increasing the risk of bias and potentially limiting the generalizability of our findings. Low response rates with surveys of clinicians is a common phenomenon^42^. However, we received responses from 35 of the 109 EIP services from all regions in England, indicating reasonable geographical coverage. A further important limitation is the absence of a patient perspective. Future work should seek the views of patients regarding the barriers and facilitators for improved clozapine treatment in EIP services.

## Conclusion

In our study, the main perceived barriers to clozapine use in EIP services by clinicians are patient concerns and physical health monitoring. National policymakers should consider the development of clinical training, improving access to resources and guidelines, and consider the streamlining of physical health monitoring to minimise barriers and facilitate the use of clozapine within EI services. National policymakers, public health agencies and NHS Trusts should consider these findings to address the underutilisation of clozapine in EIP services across England and internationally to improve patient outcomes in treatment-resistant psychosis.

## Data Availability

Authors had free access to the anonymised study data. The data that support the findings of this study are available from the corresponding author, E.O., upon reasonable request.

## References

[1] Howes OD (2017). Treatment-Resistant Schizophrenia: Treatment Response and Resistance in Psychosis (TRRIP) Working Group Consensus Guidelines on Diagnosis and Terminology. Am. J. Psychiatry.

[2] Oloyede E (2021). There Is Life After the UK Clozapine Central Non-Rechallenge Database. Schizophr Bull..

[3] Whiskey E (2021). An evaluation of the variation and underuse of clozapine in the United Kingdom. Acta Psychiatr Scand..

[4] Oloyede E (2022). Clozapine haematological monitoring for neutropenia: a global perspective. Epidemiol. Psychiatric Sci..

[5] Stokes I (2020). Prevalence of treatment resistance and clozapine use in early intervention services. BJPsych. Open.

[6] Blackman G. et al. Reducing the Risk of Withdrawal Symptoms and Relapse Following Clozapine Discontinuation—Is It Feasible to Develop Evidence-Based Guidelines? *Schizophrenia Bull*. 2021. 10.1093/schbul/sbab103 PMC8781383 34651184

[7] Pandarakalam JP (2019). The art of clozapine therapy and “clozaphobia”. BMJ.

[8] Cetin M (2014). Clozaphobia: Fear of Prescribers of Clozapine for Treatment of Schizophrenia. Klinik Psikofarmakoloji Bülteni-Bull. Clin. Psychopharmacol..

[9] Hodge K, Jespersen S (2008). Side-effects and treatment with clozapine: a comparison between the views of consumers and their clinicians. Int. J. Ment. Health Nurs..

[10] Farooq S, Choudry A, Cohen D, Naeem F, Ayub M (2019). Barriers to using clozapine in treatment-resistant schizophrenia: systematic review. BJPsych. Bull..

[11] Verdoux H, Quiles C, Bachmann CJ, Siskind D (2018). Prescriber and institutional barriers and facilitators of clozapine use: A systematic review. Schizophr. Res..

[12] Cirulli G (2005). Clozapine prescribing in adolescent psychiatry: survey of prescribing practice in in-patient units. Psychiatric Bull..

[13] Paranthaman R, Baldwin RC (2006). Survey of clozapine use by consultant old age psychiatrists. Psychiatric Bull..

[14] Singh SP (2010). Early intervention in psychosis. British J. Psychiatry.

[15] Siskind D (2022). Rates of treatment-resistant schizophrenia from first-episode cohorts: systematic review and meta-analysis. British J. Psychiatry.

[16] Nikolić N, Hill K, Campbell E, Wickramasinghe V, Whale R (2021). Early access to clozapine in Early Intervention in Psychosis: Hope vs reality. A mixed method service analysis. Early Interv. Psychiatry.

[17] Psychiatrists RCo. The Quality Standards for Early Intervention in Psychosis Services (2nd edition). 2021. https://www.rcpsych.ac.uk/docs/default-source/improving-care/ccqi/quality-networks/early-intervention-in-psychosis-teams-(eipn)/quality-standards-for-eip-services-2nd-edition.pdf?sfvrsn=131a6e4e_2 (accessed 31st May 2022).

[18] Nielsen J, Nielsen RE, Correll CU (2012). Predictors of clozapine response in patients with treatment-refractory schizophrenia: results from a Danish Register Study. J. Clin Psychopharmacol..

[19] Eysenbach G (2004). Improving the Quality of Web Surveys: The Checklist for Reporting Results of Internet E-Surveys (CHERRIES). J. Med. Internet Res..

[20] Oloyede E (2022). Clozapine for treatment resistance in early psychosis: a survey of UK clinicians’ training, knowledge and confidence. Therap. Adv. Psychopharmacol..

[21] Kelly DL, Freudenreich O, Sayer MA, Love RC (2018). Addressing barriers to clozapine underutilization: a national effort. Am. Psychiatric Assoc..

[22] Dvalishvili M, Miller BJ, Surya S (2021). Comfort Level and Perceived Barriers to Clozapine Use: Survey of General Psychiatry Residents. Acad. Psychiatry.

[23] Cotes RO (2022). A Comparison of Attitudes, Comfort, and Knowledge of Clozapine Among Two Diverse Samples of US Psychiatrists. Community Ment. Health J..

[24] Team R. C. R: A language and environment for statistical computing.: R Foundation for Statistical Computing, Vienna, Austria. 2022.

[25] Braun, V. & Clarke, V. Successful qualitative research: A practical guide for beginners. (SAGE Publishing, 2013).

[26] Thien K, O’Donoghue B (2019). Delays and barriers to the commencement of clozapine in eligible people with a psychotic disorder: a literature review. Early Interv.Psychiatry.

[27] Zheng S, Lee J, Chan SKW (2022). Utility and Barriers to Clozapine Use: A Joint Study of Clinicians’ Attitudes From Singapore and Hong Kong. J. Clin. Psychiatry.

[28] Freudenreich O, Henderson DC, Sanders KM, Goff DC (2013). Training in a clozapine clinic for psychiatry residents: a plea and suggestions for implementation. Acad. Psychiatry.

[29] Joober R, Boksa P (2010). Clozapine: a distinct, poorly understood and under-used molecule. J. Psychiatry Neurosci..

[30] Singh B, Hughes AJ, Roerig JL (2020). Comfort Level and Barriers to the Appropriate Use of Clozapine: a Preliminary Survey of US Psychiatric Residents. Acad. Psychiatry.

[31] Parkes S, Mantell B, Oloyede E, Blackman G (2022). Patients’ Experiences of Clozapine for Treatment-Resistant Schizophrenia: A Systematic Review. Schizophrenia Bulletin Open.

[32] Brodeur S (2022). Association between previous and future antipsychotic adherence in patients initiating clozapine: real-world observational study. British J. Psychiatry.

[33] Taylor D (2021). Point-of-care measurement of clozapine concentration using a finger-stick blood sample. J. Psychopharmacol..

[34] Atkins M, McGuire P, Balgobin B, Desouza N, Taylor D (2022). Haematological point of care testing for clozapine monitoring. J. Psychiatr. Res..

[35] Schulte PFJ, Bogers J, Bond-Veerman SRT, Cohen D (2020). Moving forward with clozapine. Acta. Psychiatr. Scand..

[36] Bachmann CJ (2017). International trends in clozapine use: a study in 17 countries. Acta. Psychiatr. Scand..

[37] Carruthers J (2016). An initiative to improve clozapine prescribing in New York State. Psychiatr. Services.

[38] Wheeler A, Humberstone V, Robinson G (2009). Outcomes for schizophrenia patients with clozapine treatment: how good does it get?. J. Psychopharmacol..

[39] Leung JG (2019). Addressing clozapine under-prescribing and barriers to initiation: a psychiatrist, advanced practice provider, and trainee survey. Int. Clin. Psychopharmacol..

[40] Atkins M (2022). Acceptability of point of care testing for antipsychotic medication levels in schizophrenia. Psychiat. Res. Commun..

[41] van der Zalm YC (2020). Delegating Clozapine Monitoring to Advanced Nurse Practitioners: An Exploratory, Randomized Study to Assess the Effect on Prescription and Its Safety. Adm. Policy Ment. Health.

[42] Wiebe ER, Kaczorowski J, MacKay J (2012). Why are response rates in clinician surveys declining?. Can. Fam. Physician.

